# The effect of glycidyl azide polymer on the stability and explosive properties of different interesting nitramines

**DOI:** 10.1039/c8ra02994f

**Published:** 2018-05-11

**Authors:** Ahmed K. Hussein, Ahmed Elbeih, Svatopluk Zeman

**Affiliations:** Institute of Energetic Materials, Faculty of Chemical Technology, University of Pardubice 53210 Pardubice Czech Republic; Military Technical College, Kobry Elkobbah Cairo Egypt elbeih.czech@gmail.com

## Abstract

Preparation of glycidyl azide polymer (GAP) and its influence on the stability and explosive properties of polymer bonded explosives (PBXs) based on several cyclic nitramines, namely β-1,3,5,7-tetranitro-1,3,5,7-tetrazocane (β-HMX), 1,3,5-trinitro-1,3,5-triazinane (RDX), ε-2,4,6,8,10,12-hexanitro-2,4,6,8,10,12-hexaazaisowurtzitane (ε-CL-20) and *cis*-1,3,4,6-tetranitrooctahydroimidazo-[4,5-*d*]imidazole (BCHMX) are discussed. Impact and friction sensitivity were determined. Combustion heat and detonation velocity of the studied samples were measured. The detonation parameters were obtained by the EXPLO 5 thermodynamic code. The compatibility between the energetic polymeric matrix and the studied nitramines was discussed following a vacuum stability test. The relationship between performance and sensitivity was studied in comparison with literature HTPB compositions. The results showed that the GAP matrix increased both the detonation velocities of its PBXs by more than 500 m s^−1^ and the heat of explosion by nearly 1.13–1.16 times in comparison to PBXs based on HTPB for each individual explosive. The compatibility of BCHMX to the GAP matrix seems to be better than that of CL-20/GAP.

## Abstract 2

The preparation of glycidyl azide polymer (GAP) and its influence on the stability and explosive properties of polymer bonded explosives (PBXs) based on several cyclic nitramines (CL-20, RDX, HMX and BCHMX) are reported and discussed.

## Graphical abstract

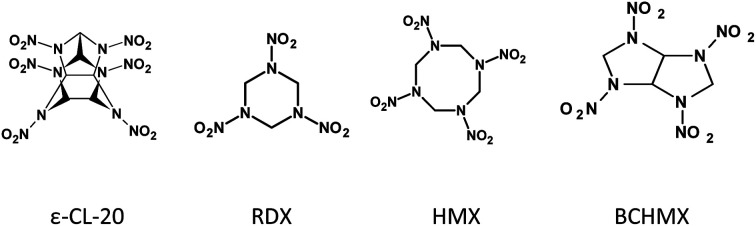

## 1. Introduction

The discovery of new energetic compounds and mixtures is a motivation for many scientists and researchers. However, Licht had proved^1^ that increasing of the explosive performance is accompanied by increasing of its sensitivity.^1–4^ He has also stated that this characteristic cannot be proved by any theory. It is also important to note that many factors, including the density of the crystals, affect the performance of the explosives. Increasing the density of the crystals can be done gradually from cyclic to polycyclic and cage structures. β-1,3,5,7-tetranitro-1,3,5,7-tetrazocane (β-HMX) and 1,3,5-trinitro-1,3,5-triazinane (RDX) are well known nitramines which have been used in several applications for more than 70 years. During the last few decades, several advanced nitramines have been reported and attracted interest from researchers, such as ε-2,4,6,8,10,12-hexanitro-2,4,6,8,10,12-hexaazaisowurtzitane (ε-CL-20) and *cis*-1,3,4,6-tetranitrooctahydroimidazo-[4,5-*d*]imidazole (BCHMX). Our research group successfully prepared BCHMX by a simple method, as described in ref. 5 and 6. The performance of BCHMX is high in comparison to the performance of traditional explosives, and its sensitivity is at same level as pentaerythritol tetranitrate (PETN).^7^ ε-CL-20 is a promising explosive; at a density of 2.04 g cm^−3^ its detonation velocity is 9800 m s^−1^.^8^ The high price of ε-CL-20 is the main problem which limits its application. These advanced explosives have been studied in comparison with traditional nitramines by several researchers.^1–4,9^ The detonation parameters of these explosives bonded by several polymeric matrices were determined.^3,4,10^ Elbeih *et al.*^10–16^ have studied the explosive properties of different plastic bonded explosives (PBXs) based on several nitramines including BCHMX. In addition, a thermo-analytical study of these PBXs was conducted by Yan *et al.*^17–19^ The penetration performance of the advanced nitramines has been studied,^20–22^ while different relationships have been presented and discussed by Zeman *et al.*^23–26^ based on the reactivity and sensitivity of the studied PBXs. BCHMX as part of a low sensitivity composition was studied by Hussein *et al.*^27–29^ From the previously mentioned research, it was concluded that the polymeric matrices have a significant influence on the different explosive properties of the studied PBXs. Glycidyl azide polymer (GAP) is a high energy polymer which has different applications, especially in composite solid rocket propellants (CSRP).^30^ It can be used as a binder or plasticizer for different types of rocket propellant and PBXs.^31–33^ The synthesis, structure and thermal behavior of the GAP binder system have been discussed in several publications.^29,34–36^ From ref. 29, 37 and 38, it was concluded that the GAP polymeric matrix is an interesting system which could have different applications with energetic materials. However, studies on the explosive properties of advanced explosives bonded by the GAP polymeric system and their compatibility remain limited in the literature. In this work, the effect of the GAP binder system on the explosive properties of several interesting explosives such as RDX, HMX, BCHMX and ε-CL-20 is presented. In addition, the compatibility of the GAP binder system with the selected explosives is discussed.

## 2. Experimental work

### 2.1. Materials

**Fig. 1:**
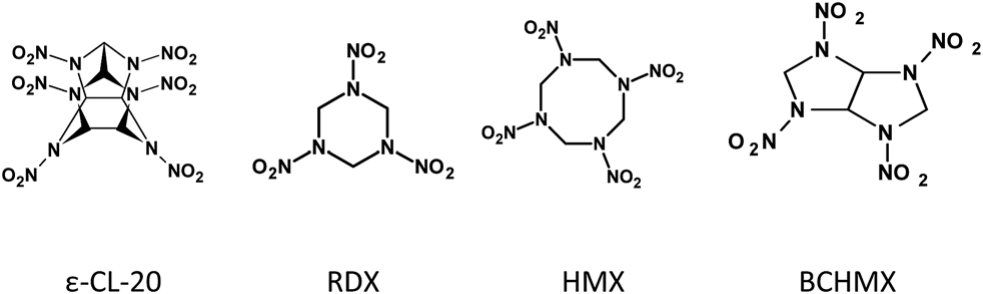
Structural formulas of the studied cyclic nitramines.

BCHMX is a bright white crystal. Similar shape and optimal particle size were obtained by recrystallization using the solvent/antisolvent (acetone/heptane) technique. HMX (β-modification, with particle size close to Class 3) was imported from Russia, RDX (with particle size close to Class 5) was a product of Dyno Nobel, and ε-CL-20 (ε-modification) was prepared in an Explosia pilot plant in the Czech Republic. The chemical structures of the studied explosives are shown in Fig. 1.

### 2.2. Preparation of GAP polymer and its PBXs

**Scheme 1:**
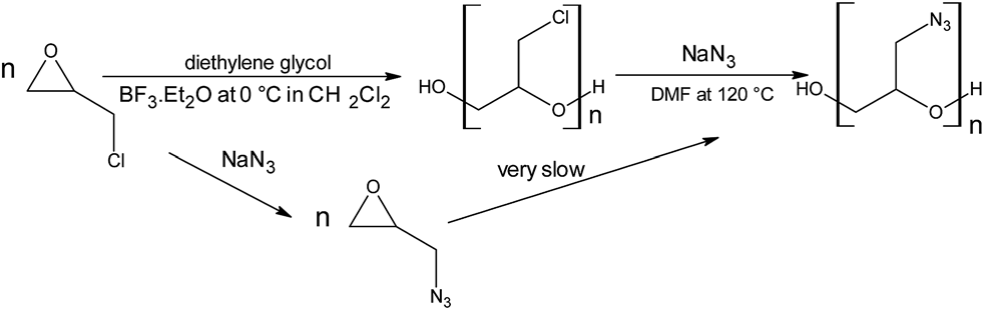
GAP preparation with diethylene glycol as the starter.

GAP was produced in our laboratories in two consecutive steps. Scheme 1 shows the GAP preparation method. The first step was the preparation of poly(epichlorohydrin) (PECH). The second step was the preparation of GAP from the PECH polymer.

#### First step

9 g of diethylene glycol (0.087 mol) was added to 360 ml of dichloromethane in a flask connected with a thermometer and CO_2_ inlet. 5 ml of cationic initiator BF_3_·OEt_2_ (boron trifluoride diethyletherate) was then injected. After 30 minutes of mixing at room temperature the solution was cooled by a mixture of water, ice, and NaCl crystals to 0 °C, after which the slow dropwise addition (0.1 ml min^−1^) of epichlorohydrin (ECH) (2.46 mol) was commenced. These operations and our own polymerization were conducted under a CO_2_ atmosphere; the gas was sourced from unprompted evaporation of “dry ice” (solid CO_2_). This dropping was continued for more than 10 hours at 0 to +3 °C in order to ensure a constant temperature and to minimise the amount of ECH in the reaction mixture. Then the mixture was left to reach room temperature. The mixing process was continued for another 6 hours. Then 700 ml of distilled water was added with ∼5 g of Na_2_CO_3_ to the mixture, and was mixed for 1 hour. The organic phase containing PECH was extracted into methylene chloride using a separating funnel, and was washed several times by distilled water until the pH was neutral. The washed organic phase was dried by adding sodium sulphate (Na_2_SO_4_), then filtered, and evaporated under vacuum to obtain 230 g of poly-epichlorohydrin.

#### Second step

230 g of poly-epichlorohydrin (PECH) was dissolved in 800 ml of dimethylformamide (DMF) in a three-necked flask fitted with a thermometer, CO_2_ inlet, and water condenser. 190 g of sodium azide was added into this solution, and then the mixture was mixed and heated to 125 °C by an oil bath for 11 hours. Then 1250 ml of water was added and mixed for 1 hour at 50 °C, followed by cooling to room temperature. 500 ml of dichloromethane was added and the phases separated using a separating funnel. The organic phase containing GAP was neutralized by washing with water. The obtained washed phase was desiccated by the addition of Na_2_SO_4_, then filtrated, and evaporated under vacuum to obtain 228 g of GAP. The theoretical yield was 243 g, and thus the yield was 93%.

The number average molecular weight (*M*_n_) was 1757 kg mol^−1^ and the weight average molecular weight (*M*_w_) was 2459 kg mol^−1^. The polydispersity index (PDI) is 1.4. These results were obtained after analysis by GPC against polystyrene standards. The viscosity of GAP was 5000 cP at 25 °C, the measured value of mg equivalents of OH per g of GAP was 0.825 mg eq. per g, and the measured nitrogen content was 40.3%.

The GAP polymeric matrix was fabricated with a BRABENDER plastograph. Water flow was circulated through the mixer jacket at 40 ± 2 °C. Mixing of the liquid ingredients, GAP, and dioctyl adipate (DOA) [obtained from ACROS Organics] was done for 20 minutes under vacuum to remove trapped air. The solid ingredient (pure explosive) was added at 40 °C over 30 minutes, followed by the addition of 1,1,1-tris(hydroxymethyl)ethane (THME) as a cross linking agent (obtained from Merck & Co.), and the curative hexamethylene diisocyanate (HMDI) (obtained from ACROS Organics). The addition was performed at 55 °C and mixed for another 30 minutes under vacuum. After completion of the mixing process, the mixture was cast under vacuum and cured at 60 ± 2 °C for seven days. The ratio of NCO/OH was 0.85. The masses of the plasticizer and the cross linking agent were 7.5 and 5.0 wt% of the total mass of the polymeric matrix respectively. The weight percentage composition of the GAP PBXs was 16 wt% GAP binder, and 84 wt% explosive. The PBXs are designated as RDX/GAP, HMX/GAP, BCHMX/GAP, and CL-20/GAP.

### 2.3. Elemental analysis

C, H, and N elements of the prepared PBXs were measured using Fisons EA-1108 CHNS–O elemental analyzer. Recalculation of the matched nitrogen content was used to obtain the formula for the individual explosives. The formulas were used as input data to calculate detonation parameters using the EXPLO 5 code.

### 2.4. Heat of combustion determination

A high pressure bomb calorimeter, model BCA 500, obtained from OZM Research, Czech Republic, was used to determine the heat of combustion of BCHMX/GAP, as well as the other PBXs. The sample was inserted into the bomb and filled with an excess of oxygen,^39^ and then ignited. The enthalpy of formation was obtained after calculation of the output data obtained from the instrument. The enthalpy of formation was used for the calculation of the detonation characteristics of the studied samples.

### 2.5. Sensitivity to impact and friction

A BAM impact sensitivity instrument with an exchangeable drop weight was used to perform standard impact tests;^40^ the volume of each tested sample was 50 mm^3^, and different weights were used as the dropping hammer. The probability of initiation was determined by Probit analysis. Only 50% probability of initiation (H50) was determined, and the results are listed in Table 1. Sensitivity to friction using standard test conditions^40^ was determined by a BAM friction test apparatus. Sensitivity to friction for the studied PBXs was detected by spreading 0.01 g of the sample on the rough surface of the porcelain plate. Change to the normal force between the porcelain pistil and the plate was applied by the use of different loads. Any sort of decomposition (such as smoke, sound or smell) was considered as a positive sample initiation. Using Probit analysis,^41^ 50% of initiations are specified and are listed in Table 1 as the friction sensitivity.

**Table 1:** Results of the experimental measurements of the studied samples, and literature data of HTPB compositions of 18% weight binder

No.	Code designation	Summary formula	Heat of combustion [J g^−1^]	Enthalpy of formation [kJ mol^−1^]	Impact sensitivity [J]	Friction sensitivity [N]
1	BCHMX	C_4_H_6_N_8_O_8_	9124	236.5	3.2^a^	88^a^
2	β-HMX	C_4_H_8_N_8_O_8_	9485	77.3	6.4^a^	95^a^
3	RDX	C_3_H_6_N_6_O_6_	9522	66.2	5.6^a^	120^a^
4	ε-CL-20	C_6_H_6_N_12_O_12_	8311	397.8	4.1^b^	69^b^
5	BCHMX/HTPB	—	13 798	—	9.6^c^	322^c^
6	HMX/HTPB	—	14 118	—	15.2^c^	>360^c^
7	RDX/HTPB	—	14 162	—	14.6^c^	>360^c^
8	CL-20/HTPB	—	13 255	—	10.8^c^	214^c^
9	BCHMX/GAP	C_4.94_H_7.89_N_8_O_7.27_	11 789	414.8	7.7	294
10	HMX/GAP	C_4.84_H_8.48_N_8_O_7.25_	11 893	256.4	11.2	338
11	RDX/GAP	C_3.67_H_7.12_N_6_O_5.39_	12 011	198.3	11.5	>360
12	CL-20/GAP	C_7.47_H_9.33_N_12_O_11.17_	10 922	597.4	8.4	247

a Value sourced from ref. 10.

b Value sourced from ref. 12.

c Value sourced from ref. 25.

### 2.6. Detonation velocity measurements

The detonation velocity of the prepared compositions was determined by the ionization copper probe method.^40^ The setup required for the measurement consists of an oscilloscope (Escort EUC-3200) with an electronic counter connected to a capacitor. The capacitor was connected to the ionization probes, which were placed inside the explosive charge. Twisted copper wire with 0.25 mm radius was used. The PBXs were cast in the form of cylinders with 200 mm length and 21 mm diameter. The measurements were determined by measuring the exact distance between the two probes, and then recording the time at which the detonation wave moved between the two probes. The twisted ionization copper was located in each charge, with the first sensor being placed at a distance of 50 mm from the surface containing the detonator. Detonator no. 8 was used as an initiator. Each sample was measured three times and the mean values (max. ±76 m s^−1^) are reported in Table 2.

**Table 2:** Detonation parameters of the studied samples, and literature data of HTPB compositions of 18% weight binder

Studied sample		Experimental			Detonation parameters calculated by Explo5		
No.	Code of samples	Density *ρ* [g cm^−3^]	Detonation velocity [m s^−1^]		Error%	Detonation pressure *P* [GPa]	Heat of Explosion *Q* [kJ kg^−1^]
			*D*_exp_	*D*_cal._			
1	BCHMX	1.79^a^	8650	8840	+2.19	33.9	6447
2	β-HMX	1.90^b^	9100	9225	+1.37	38.0	6075
3	RDX	1.76^b^	8750	8718	−0.40	32.1	6085
4	ε-CL-20	1.98^b^	9473	9407	−0.60	41.7	6455
5	BCHMX/HTPB	1.56^c^	7746	7593	−1.97	21.2	5744
6	HMX/HTPB	1.57^c^	7812	7627	−2.36	21.3	5453
7	RDX/HTPB	1.52^c^	7526	7449	−1.02	20.1	5453
8	CL-20/HTPB	1.63^c^	8167	7919	−3.03	23.7	5786
9	BCHMX/GAP	1.62	8292	8261	−0.37	28.6	6658
10	HMX/GAP	1.64	8384	8313	−0.85	28.4	6297
11	RDX/GAP	1.59	8074	8099	+0.31	26.2	6152
12	CL-20/GAP	1.73	8676	8482	−2.23	32.1	6559

a Value sourced from ref. 7.

b Value sourced from ref. 12.

c Value sourced from ref. 16.

### 2.7. Calculation of the detonation properties

The theoretical detonation properties (detonation velocity, *D*, heat of detonation, *Q*, and detonation pressure, *P*) of the studied compositions, in addition to the individual nitramines, were calculated by the EXPLO5 code version 5.04.^42^ The following BKWN set of parameters was used: *α* = 0.5, *β* = 0.176, *κ* = 14.71, *Θ* = 6620.^42^ The heat of explosion is the heat released at a constant volume of explosion and is determined by subtracting the heats of formation of the explosive (reactants) from the sum of the heats of formation of the detonation. The theoretical calculation and the error between the experimental results are listed in Table 2.

### 2.8. Vacuum stability test

The compatibility of the PBXs was tested by a vacuum stability test (VST) with a modernized STABIL 16-Ex apparatus^43^ (manufactured by OZM Research; the original apparatus is described in ref. 44). The measurement procedures have previously been reported.^23,25,45,46^ The amount of sample used for a measurement was 2 g. The duration of the test was 40 hours. The temperature for the isothermal measurements was chosen to be 120 °C. Samples in evacuated glass test tubes were placed into the heating block and heated to the desired temperature.

The increase in pressure was continuously measured by pressure transducers inside the glass tubes. This test determines the gas pressure evolution per 1 g of sample in dependence with time. A linear relationship was observed based on each curve being tested isothermally for 60–2400 minutes. The slope of these lines, *k*, corresponds to the reaction velocity of gaseous product evolution in a zero-order reaction,^23,25,45,46^ and in this case, the slope *k* expresses the specific rate constant (in kPa g^−1^ min^−1^).

## 3. Results and discussion

**Fig. 2:**
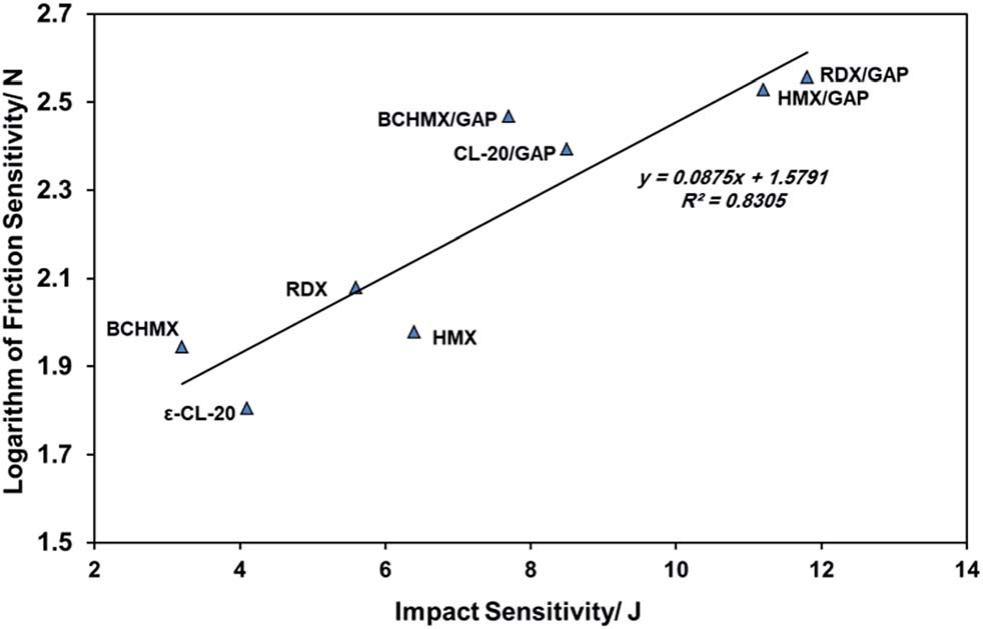
Semi-logarithmic relationship between friction and impact sensitivities.

**Fig. 3:**
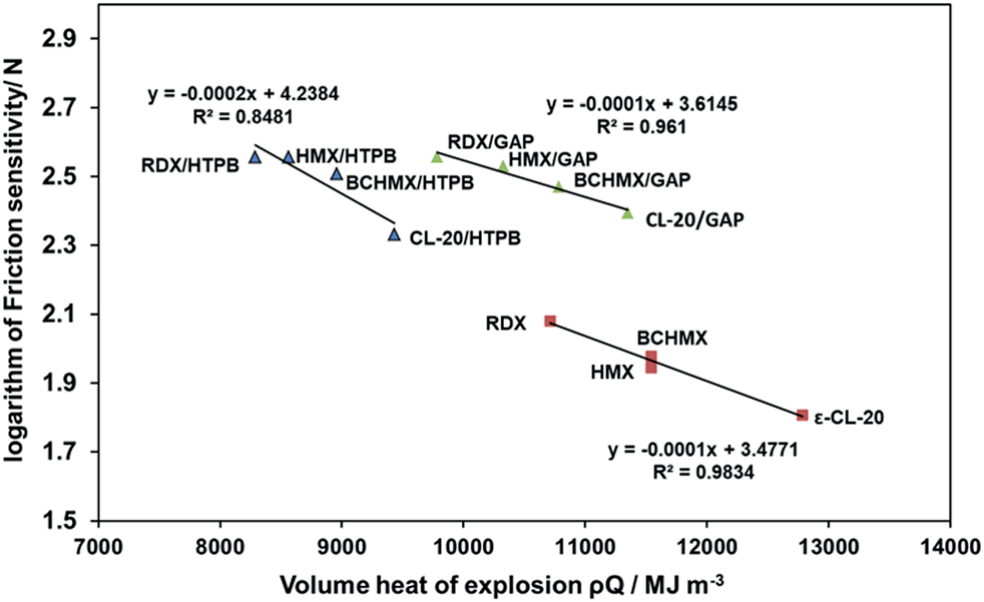
Semi-logarithmic relationship between friction sensitivity and volume heat of explosion.

The performance of an explosive material has a significant effect on its impact and friction sensitivity, which is attested to by recently published literature,^47^ that is to say, high performance of an explosive is accompanied by an increase in explosive sensitivity. However, modern techniques can be used to combine a high performance value with a reduction in sensitivity. In order to study the sensitivity of the studied samples, a comparison between the impact sensitivity (initiation due to uniaxial compression) and the friction sensitivity (initiation due to shear slide of a fixed volume) of all the studied samples is shown in Fig. 2 It is obvious that the sensitivity of the nitramines in the GAP matrix have been changed significantly. Nevertheless, BCHMX/GAP and CL-20/GAP still have relatively high sensitivity compared to HMX/GAP and RDX/GAP. It is also important to mention that the sensitivity to friction of RDX is improved by its incorporation into GAP matrix. In the sensitivity evaluation, comparison with performance should be represented. Thus, the relation between the volume heat of detonation (the product of loading density and the volume explosion heat *ρQ*/MJ m^−3^) and the logarithm of impact sensitivity was studied in ref. 48, while the same relation based on the logarithm of friction sensitivity is presented in Fig. 3. Three independent lines are presented in Fig. 3. One of them connects the pure nitramines, and the performance of both BCHMX and HMX is approximately the same despite the higher density of HMX. The other two lines connect the PBXs based on HTPB on the left, and the PBXs based on the GAP binder on the right. It is clear that all of the studied PBXs based on the GAP binder have a higher volume heat of explosion compared to the PBXs based on the HTPB binder. In addition, PBXs based on BCHMX have a higher heat of explosion than PBXs based on RDX and HMX for the same individual binder. These results are clear due to the high heat of formation of BCHMX compared to HMX (see Table 2). Also, due to the presence of the energetic GAP matrix, the heat of explosion of BCHMX/GAP is 1.15 times higher than that of BCHMX/HTPB.

ε-CL-20 based PBXs have the highest friction sensitivity of all the studied samples. The depicted relationship attests to a clear link between sensitivity and performance, which has been pointed out by Licht,^1^ who has reported that increasing the performance of an explosive is accompanied by an increase in its sensitivity.^1–4^

**Fig. 4:**
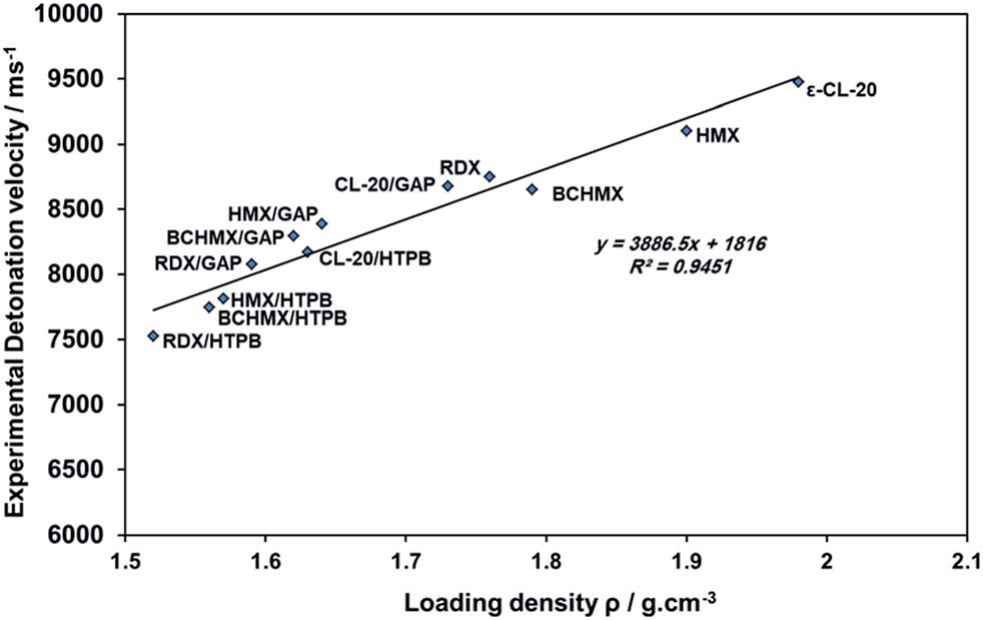
The relationship between the density and the detonation velocity of all the studied explosives.

Fig. 4 represents the well-known relationship between experimental loading density and velocity of detonation. The velocities of detonation of the studied GAP bonded explosives are higher than those of HTPB bonded explosives of each corresponding explosive. CL-20/GAP is in the same range as pure RDX and BCHMX. Also, BCHMX/GAP has a higher detonation velocity than CL-20/HTPB, which means that BCHMX/GAP might be able to replace the expensive composition CL-20/HTPB, given its performance. BCHMX/GAP has a higher detonation velocity than BCHMX/HTPB by nearly 500 m s^−1^. It means that GAP binder has a greater positive influence on performance compared to HTPB binder. Also, the velocity of detonation of BCHMX/GAP is considered to be between the detonation velocity values of RDX/GAP and HMX/GAP.

**Fig. 5:**
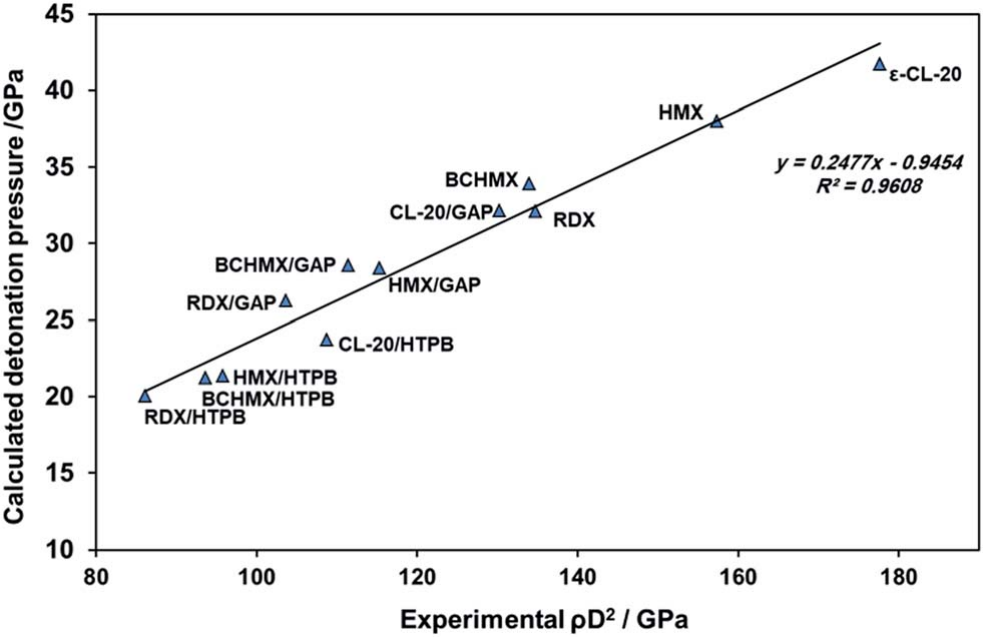
Relationship between calculated detonation pressure and the square of the experimental detonation velocity multiplied by the loading density.

Regarding the theory of detonation, the detonation pressure of the energetic materials could be represented by the product of the square of the detonation velocities and the loading densities. Fig. 5 presents a comparison between the calculated detonation pressures and the mentioned measured products. The good fit of this relationship demonstrates a good match between the calculated results by EXPLO5 and the measured data. The calculated detonation pressures of all the studied samples based on GAP binder are higher than the samples based on HTPB binder.

In order to check the compatibility between the polymeric matrix and the nitramines practically, a vacuum stability test was conducted to determine the amount of gas that evolved from the compositions, which was compared to that for the pure nitramines. From the obtained results, the amount of the gas liberated from RDX was the highest compared to those of the other nitramines in this study. In addition, ε-CL-20 is considered to be more stable than pure BCHMX, while HMX is the most stable explosive of the studied pure nitramines. Nevertheless, these characteristics are changed in the presence of the GAP matrix; it was observed that the amount of gas liberated from CL-20/GAP reached 12 ml g^−1^, while BCHMX/GAP produced half the amount liberated from CL-20/GAP. RDX/GAP produced less gas than both BCHMX/GAP and CL-20/GAP, while HMX/GAP remained the most stable composition compared with the studied compositions. Also, it is clear that the presence of the GAP matrix significantly increased the amount of gas that evolved from the individual explosives, however, the chemical structure and the physical stability of the nitramine plays a role in the presence of GAP.^29^

**Fig. 6:**
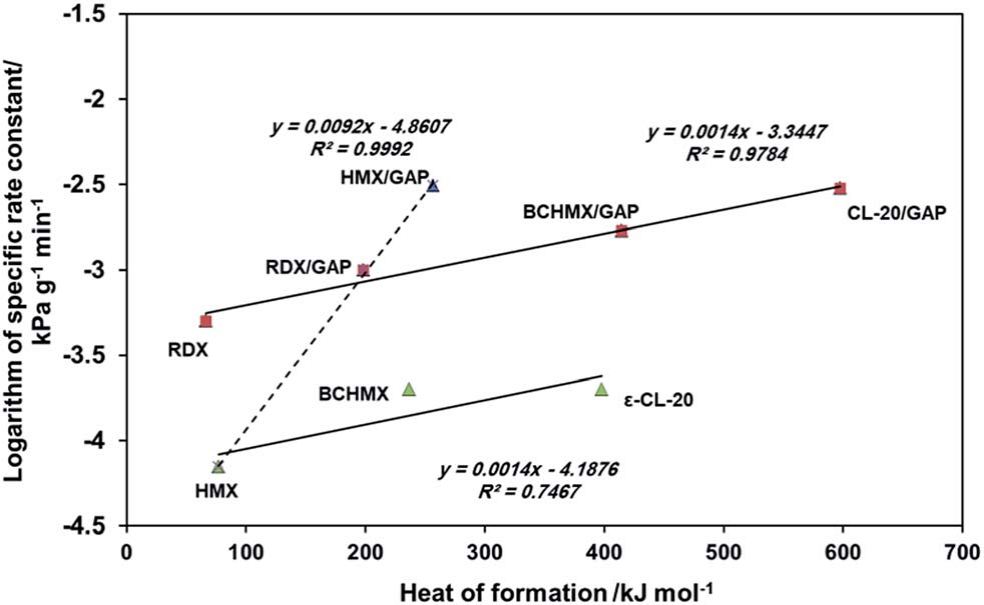
Relationship between the logarithm of the specific rate constant and heat of formation of the studied PBXs and pure nitramines.

To check the possibility of linking the results of the vacuum stability test with the energetic content of the studied explosives, Fig. 6 presents the relationship between the heat of formation and the logarithm of the specific rate constant (*i.e.* the slope from Table 3). This figure clarifies the good evidence for the link between increases in reactivity and the increase of the energy content of the energetic material molecules. The line connecting the GAP nitramines with pure RDX in Fig. 6 represents the order of stability of these studied explosives. It is observed that the BCHMX/GAP explosive is more stable than CL-20/GAP, while it is less stable than both pure RDX and the composition RDX/GAP. Pure HMX is the most stable explosive of all of the studied samples. These results are also confirmed by the amount of gas evolved per gram of sample (see Table 3).

**Table 3:** Summary of the vacuum stability test results of the nitramine explosives studied; with exposure to 120 °C for 40 hours

Abbreviation	Gas evolved per gram [ml g^−1^]	Slope [kPa g^−1^ min^−1^]	Intersection [kPa]	*R* ^2^
BCHMX	0.117	0.0002	0.5641	0.9811
	0.131	0.0002	0.5831	0.9941
HMX	0.041	0.00007	0.2707	0.9174
	0.036	0.00007	0.6728	0.9013
RDX	0.367	0.0005	1.7608	0.9144
	0.34	0.0005	2.0799	0.9193
ε-CL-20	0.09	0.0001	0.4599	0.9506
	0.089	0.0002	0.6334	0.957
BCHMX/GAP	6.746	0.0019	0.0693	0.9990
	6.496	0.0017	0.0275	0.9990
HMX/GAP	1.291	0.0036	0.6122	0.9927
	1.225	0.0031	0.8656	0.9939
RDX/GAP	4.374	0.001	0.0932	0.9961
	3.673	0.001	0.1391	0.9947
CL20/GAP	12.358	0.003	2.3946	0.9552
	11.894	0.0028	2.2903	0.954

## 4. Conclusions

Glycidyl azide polymer used for the preparation of PBXs to a certain extent decreases the sensitivity of the studied nitramines; nevertheless, the PBXs filled with BCHMX and ε-CL-20 still have the character of a relatively sensitive explosive – their impact sensitivity is lower than 10 J. According to expectation, the GAP matrix has a positive influence that increases the detonation characteristics of nitramine PBXs; compared with classical non-energetic binders, the heat of explosion of these PBXs are 1.13–1.16 times higher (that of the BCHMX/GAP is 1.15 times higher than those with the HTPB binder). The explosion heat of BCHMX/GAP appears to be the highest among the studied PBXs, while in terms of experimental detonation velocity, the highest value was obtained for CL-20/GAP (8676 m s^−1^). The calculated explosive parameters, obtained by means of the EXPLO5 code, are in a good agreement with the experimental results with an error less than 2.5%. Concerning the influence of GAP on the mechanical sensitivities of the PBXs studied, it is possible to state that this binder has a slightly lower desensitizing effect in comparison to the HTPB binder. Also, an inverse proportionality existed between the performance and mentioned sensitivities of the studied plastic bonded explosives. Vacuum stability tests at 120 °C showed that BCHMX has a higher resistance against the influence of GAP than does ε-CL-20, while HMX has the best compatibility. The mutual comparison between the specific rate constants obtained from this test and the heat of formation of the studied explosives, clearly confirmed a link between the increase of initiation reactivity with an increase in energy content in the molecules of energetic materials.

## Conflicts of interest

There are no conflicts to declare.

## Supplementary Material
